# Prehospital Point-of-Care Lactate as a Predictor of Early Operative and Emergency Interventions in Trauma Patients: A Systematic Review

**DOI:** 10.7759/cureus.100559

**Published:** 2026-01-01

**Authors:** Mosab A Alabas, Nasser M Hakami, Fahad Y Azyabi, Haneen Y Alsayed, Maen A Idris, Nasser F Alshanbari, Naif N Althagafi, Ahmed H Alabdele, Mohammad M Alfaidi, Abdulaziz A Alabdulrahman, Jerayed K Aljerayed

**Affiliations:** 1 Department of Emergency Medicine, Aseer Central Hospital, Abha, SAU; 2 Department of Emergency Medicine, South Al-Qunfudah Hospital, Al-Qunfudah, SAU; 3 Department of Urology, King Salman bin Abdulaziz Medical City, Al-Madina, SAU; 4 Faculty of Medicine, King Abdulaziz University, Jeddah, SAU; 5 College of Medicine, King Abdulaziz University, Jeddah, SAU; 6 College of Medicine, King Saud University, Riyadh, SAU; 7 College of Medicine, King Saud bin Abdulaziz University for Health Sciences, Riyadh, SAU

**Keywords:** early operative intervention, emergency surgery, lactate measurement, normotensive patients, point-of-care lactate, prehospital care, resuscitative care, risk stratification, surgical preparedness, trauma

## Abstract

Early identification of trauma patients requiring immediate operative or emergency intervention remains a major challenge in the prehospital setting. Traditional physiological parameters, such as systolic blood pressure and shock index, may fail to detect occult hypoperfusion, particularly in patients with normal blood pressure (normotensive). Prehospital point-of-care (POC) lactate measurement, which allows rapid bedside assessment of blood lactate levels, has emerged as a potential biomarker to improve early risk stratification and guide timely surgical preparedness. Evidence from observational studies suggests that elevated prehospital lactate is consistently associated with an increased likelihood of early invasive management, including emergency surgery, interventional radiology, and resuscitative care. Lactate appears to offer superior or complementary predictive performance compared with traditional physiological measures, particularly in normotensive trauma patients, and is most strongly correlated with immediate operative intervention within six hours of injury. Despite these promising findings, a small number of studies and the absence of prospective lactate-guided interventional trials limit the current evidence. Integrating prehospital lactate measurement into trauma triage and decision-making algorithms may enhance early recognition of patients requiring urgent operative care, but further research is needed to determine whether this approach improves operative timing and clinical outcomes.

## Introduction and background

Traumatic injury remains a leading cause of mortality and morbidity worldwide, particularly among young and working-age populations [1,2]. Hemorrhage and unrecognized tissue hypoperfusion are common preventable causes of early death following trauma, often occurring within the first few hours after injury [3,4]. Early identification of patients at high risk for deterioration and in need of urgent operative or resuscitative intervention is therefore essential [1-5]. Prehospital triage decisions play a critical role in this process, influencing transport destination, trauma team activation, and early mobilization of surgical and transfusion resources [5,6].

Traditional prehospital assessment relies on physiological parameters such as systolic blood pressure, heart rate, respiratory rate, and level of consciousness [6]. However, these vital signs may remain within normal ranges during early hemorrhagic shock due to compensatory mechanisms, leading to under-recognition of occult hypoperfusion [6,7]. This limitation has prompted interest in adjunctive biomarkers that more accurately reflect global tissue perfusion and metabolic stress in the prehospital setting [7,8].

Blood lactate is a well-established marker of anaerobic metabolism and tissue hypoxia, and advances in point-of-care (POC) testing now allow rapid lactate measurement before hospital arrival [1-4,8]. Prehospital lactate has been proposed as a tool to enhance early risk stratification, identify patients with occult shock, and support more informed triage and resuscitation decisions [5-8]. Observational studies suggest that elevated prehospital lactate is associated with an increased likelihood of blood transfusion, resuscitative care, and emergency operative or interventional procedures, sometimes outperforming traditional vital signs, particularly in normotensive patients [9-12].

Despite growing interest, the role of prehospital lactate in predicting early operative or emergency interventions has not been systematically synthesized [1-8]. Variations in study design, patient selection, timing of lactate measurement, and outcome definitions have limited translation into standardized clinical algorithms. This systematic review aims to critically evaluate and qualitatively synthesize the evidence on prehospital lactate as a predictor of early operative or emergency interventions in trauma patients, focusing on measurements obtained before hospital arrival and clinically relevant outcomes within the early hours of admission.

## Review

Methodology

Literature Search Strategy

A systematic review was conducted in accordance with the Preferred Reporting Items for Systematic Reviews and Meta-Analyses (PRISMA) guidelines [13]. A comprehensive search was performed across PubMed, Scopus, Web of Science, and the Cochrane Library from database inception to the final search date (Appendix 1). Keywords and controlled vocabulary related to trauma, prehospital care, lactate measurement, and early operative or emergency outcomes were used. Searches were limited to human studies, excluding reviews, editorials, conference abstracts without full data, protocols, and non-original publications. No restrictions were applied regarding publication year, language, or geographic location.

Eligibility Criteria

Studies were included if they involved adult trauma patients in the prehospital setting with POC lactate measurement and reported early operative or emergency outcomes, such as emergency surgery, interventional radiology for hemorrhage control, massive transfusion, or resuscitative care within hours of hospital arrival. Prospective or retrospective observational studies and diagnostic or prognostic accuracy studies were eligible. Comparators could include prehospital vital signs, shock index, physiological scores, or triage criteria. Studies were excluded if lactate was measured only after hospital admission, focused on non-trauma populations, or had outcomes unrelated to operative or emergency interventions.

Study Selection

Records were imported into reference management software, and duplicates were removed. Titles and abstracts were screened to exclude irrelevant studies. Full texts of potentially eligible articles were assessed against inclusion and exclusion criteria, focusing on timing of lactate measurement, prehospital setting, and relevant outcomes. Disagreements were resolved by discussion and consensus.

Data Extraction and Quality Appraisal

Data were extracted using a standardized table including study design, sample size, trauma population characteristics, timing and method of lactate measurement, comparator variables, outcome definitions, timeframes, and predictive performance metrics. Risk of bias was assessed using the Quality Assessment of Diagnostic Accuracy Studies-2 (QUADAS-2) tool across four domains: patient selection, index test, reference standard, and flow and timing [14]. Overall risk-of-bias assessments informed the qualitative synthesis. Due to study heterogeneity and limited numbers, no meta-analysis was performed, and findings were synthesized narratively.

Results

Study Selection

**Figure 1:**
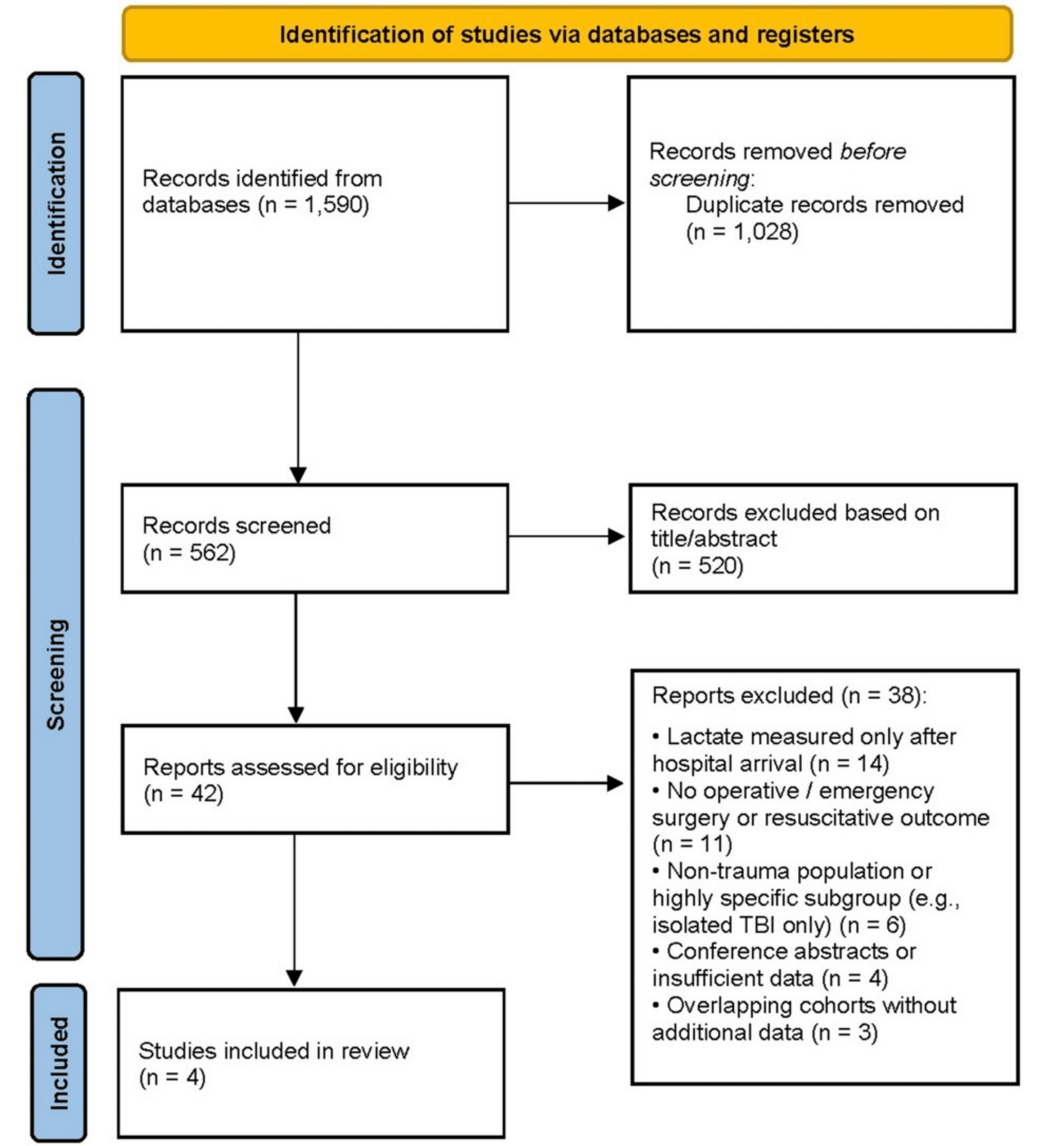
PRISMA flow diagram illustrating the study selection process PRISMA flow diagram depicting the identification, screening, eligibility assessment, and inclusion of studies in the systematic review, including records identified through database searching, duplicates removed, full-text articles assessed for eligibility, and studies included in the final qualitative and quantitative synthesis [13]. PRISMA: Preferred Reporting Items for Systematic Reviews and Meta-Analyses; TBI: traumatic brain injury

The database search identified 1,590 records, with 562 unique articles screened after removing duplicates. Title and abstract screening excluded 520 records, primarily due to the absence of prehospital lactate measurement, non-trauma populations, lack of operative or emergency outcomes, or non-original publication types. A full-text review of 42 articles led to the exclusion of 38 studies for reasons including lactate measured only after hospital arrival, no relevant outcomes, non-trauma populations, insufficient data, or overlapping cohorts. Four studies [1-4] met all inclusion criteria and were included in the qualitative synthesis (Figure 1). Due to heterogeneity in study design and outcomes, no meta-analysis was performed.

Characteristics of Included Studies

The four studies assessed prehospital POC lactate in adult trauma patients across different emergency medical service (EMS) systems and countries. Two studies were conducted in the United States [1,3], one in Japan [4], and one in the United States cohort of normotensive trauma patients [2]. Study designs included prospective, retrospective, and combined approaches, with sample sizes ranging from 314 to over 1,100 patients. Lactate was measured at the scene or during transport before hospital arrival, and comparator variables included traditional vital signs such as systolic blood pressure, heart rate, respiratory rate, Glasgow Coma Scale, and shock index [1,2,4]. Outcomes focused on early trauma-related interventions, including emergency surgery, interventional radiology, blood transfusion, and resuscitative care, within six to 24 hours of emergency department arrival (Table 1) [1,2,4].

**Table 1:** Summary of characteristics of the included studies Summary of included studies evaluating prehospital point-of-care (POC) lactate as a predictor of early operative or emergency interventions in trauma patients. EMS: emergency medical service; SBP: systolic blood pressure; HR: heart rate; RR: respiratory rate; GCS: Glasgow Coma Scale; SI: shock index; ED: emergency department; IR: interventional radiology; AUC: area under the receiver operating characteristic curve; OR: odds ratio; MODS: multiple organ dysfunction syndrome; ALS: advanced life support. The table presents study design, clinical setting, sample size, trauma population characteristics, timing and type of lactate measurement, comparator variables, outcome definitions, outcome timeframes, inclusion of operative or emergency surgery within outcomes, key predictive metrics, main findings, and relevant methodological notes for each study.

Author	Country	Study Design	Setting	Sample Size	Trauma Population	Lactate Measurement (Timing & Type)	Comparator(s)	Outcome Definition	Outcome Timeframe	Operative/Emergency Surgery Included	Predictive Metrics	Key Findings	Methodological Notes
Guyette et al. [1]	USA / Canada (ROC consortium)	Prospective multicenter observational diagnostic study	Prehospital ground EMS → Level I/II trauma centers	387 (SBP 71–100 mmHg cohort)	Adult blunt and penetrating trauma patients with prehospital SBP ≤100 mmHg	POC venous lactate measured at scene during IV insertion using a handheld device (Lactate Pro); EMS and hospital teams blinded	SBP (≤90 mmHg), shock index (SI)	Need for resuscitative care defined as ≥5 units blood transfusion, hemorrhage control intervention (laparotomy, thoracotomy, pelvic fixation, interventional radiology (IR) embolization), or death	≤6 hours after ED arrival	Yes (laparotomy, thoracotomy, pelvic fixation, IR embolization included)	AUC: lactate 0.78 vs SBP 0.59 vs SI 0.66; sensitivity 93% (lactate ≥2.5 mmol/L) vs 67% (SBP ≤90)	Prehospital lactate significantly outperformed SBP and shock index in predicting early resuscitative care; the strongest predictive value in lactate range 2.5–3.9 mmol/L	Composite outcome; focused on hypotensive/near-hypotensive patients; emergency surgery not isolated; lactate values blinded to clinicians
St. John et al. [2]	USA	Secondary analysis of a prospective observational cohort	Prehospital advanced life support (ALS) ground EMS → Level I trauma center	314	Adult normotensive trauma patients (blunt and penetrating); SBP >100 mmHg	POC capillary blood lactate measured at IV placement using handheld Lactate Pro device	Shock index (HR/SBP)	Need for resuscitative care defined as death in ED, emergent surgery or interventional radiology within 6 h, or ≥5 units blood transfusion within 6 h	≤6 hours after ED arrival	Yes (emergent surgery and IR explicitly included)	AUC 0.716 (lactate) vs 0.631 (shock index), p = 0.125; sensitivity 74.6%, specificity 53.4% at ≥2.5 mmol/L	Prehospital lactate predicted need for early resuscitative care in normotensive trauma patients; higher lactate associated with increased odds of emergent intervention	Composite outcome; surgery not isolated; hypotensive patients excluded; single-center receiving hospital; device no longer commercially available
Guyette et al. [3]	USA	Retrospective observational cohort study	Prehospital air medical transport → Level I trauma center	1168	Adult trauma patients (mixed blunt and penetrating)	POC serum lactate measured during air transport	Prehospital vital signs, shock index, respiratory distress, altered mental status	In-hospital mortality, emergent surgery, and multiple organ dysfunction syndrome (MODS)	During index hospitalization (emergent surgery within early hospital course)	Yes (emergent surgery included as secondary outcome)	AUC for surgery improved from 0.68 to 0.71 with lactate (p = 0.02); odds ratio (OR) for surgery 1.13 per 1 mmol/L lactate increase	Higher prehospital lactate independently associated with increased need for emergent surgery and mortality; lactate improved predictive performance beyond vital signs	Emergent surgery not primary endpoint; timing not strictly limited to 6–24 h; air-medical population may limit generalizability
Fukuma et al. [4]	Japan	Prospective observational study with retrospective analysis and external validation	Prehospital EMS → Level I trauma center	435 (derivation) + 85 (validation)	Adult trauma patients (mixed blunt and penetrating)	Point-of-care (POC) blood lactate measured at the scene; repeated on emergency department (ED) arrival	Prehospital physiological variables (systolic blood pressure (SBP), heart rate (HR), respiratory rate (RR), Glasgow Coma Scale (GCS))	Immediate intervention for hemorrhage, defined as surgical intervention, radiological intervention, and/or blood transfusion	≤24 hours after hospital arrival	Yes (surgical and radiological hemorrhage control included as part of composite outcome)	Area under the curve (AUC) 0.882 (physiology + lactate) vs 0.837 (physiology alone), p = 0.0073	Addition of prehospital lactate significantly improved the prediction of immediate hemorrhage-related interventions; rising lactate from scene to ED strongly associated with increased intervention probability	Outcome is composite; emergency surgery not isolated; device type not specified

Methodological Quality

Overall risk of bias was low to moderate. Patient selection was appropriate in all studies, with consecutively or prospectively enrolled cohorts in three studies [1,2,4] and a retrospective cohort in one study [3]. Prehospital lactate measurement was consistently performed before hospital arrival, with blinding reported in one study [1]. Reference standards were objectively defined, and outcomes were assessed within early predefined timeframes in three studies [1,2,4]. One study [3] had an unclear timing for operative interventions, resulting in an overall moderate risk of bias (Table 2).

**Table 2:** Summary of methodological quality assessment of included studies Risk-of-bias assessment of included studies using the QUADAS-2 tool [14]. EMS: emergency medical service; POC: point-of-care; SBP: systolic blood pressure; IR: interventional radiology; ED: emergency department. Judgments of risk (low, unclear, or moderate) are provided for four domains: patient selection, index test (prehospital lactate), reference standard (operative or emergency intervention), and flow and timing (temporal alignment of lactate measurement and outcomes). Justifications summarize the rationale for each judgement, and the overall risk of bias reflects the combined assessment across all domains for each study.

Study	Patient Selection (Judgement + Justification)	Index Test: Prehospital Lactate (Judgement + Justification)	Reference Standard (Judgement + Justification)	Flow & Timing (Judgement + Justification)	Overall Risk of Bias
Guyette et al. [1]	Low risk: Prospective, multicenter enrollment of trauma patients with predefined systolic blood pressure (SBP) criteria reduced selection bias and improved representativeness.	Low risk: Prehospital lactate measured using a POC device at the scene; EMS and hospital clinicians blinded to lactate values, reducing bias.	Low risk: Resuscitative care included clearly defined emergency operative and interventional procedures (laparotomy, thoracotomy, pelvic fixation, interventional radiology (IR) embolization).	Low risk: Outcomes assessed within a strict ≤6-hour timeframe after emergency department (ED) arrival, ensuring appropriate temporal alignment.	Low
St. John et al. [2]	Low risk: Trauma patients prospectively enrolled with explicit inclusion of normotensive patients; exclusion criteria were predefined and appropriate.	Low risk: Prehospital lactate obtained prior to hospital arrival using POC testing, measured before outcome determination.	Low risk: Reference standard included emergency surgery, IR, or massive transfusion, all clinically relevant and objectively recorded.	Low risk: Outcomes assessed within 6 hours of ED arrival, consistent with immediate operative intervention.	Low
Guyette et al. [3]	Low risk: Large consecutive cohort of air-medical trauma patients with no evidence of selective inclusion or inappropriate exclusions.	Low risk: Prehospital lactate measured during transport prior to hospital arrival and before outcomes were known.	Low risk: Emergent operative intervention clearly defined; analyzed as a secondary outcome rather than a primary endpoint.	Unclear risk: Timing of emergent surgery relative to ED arrival not strictly bounded (e.g., ≤6 or ≤24 hours).	Moderate
Fukuma et al. [4]	Low risk: Consecutive trauma patients included with clearly defined eligibility criteria; no inappropriate exclusions reported.	Low risk: Prehospital lactate measured at the scene using point-of-care (POC) testing before outcomes were known; interpretation not influenced by clinical decisions.	Low risk: Reference standard of immediate hemorrhage intervention (surgery, radiological intervention, or transfusion) was clinically appropriate and objectively defined.	Low risk: Lactate measured prehospital and outcomes assessed within a clearly defined 24-hour window, minimizing misclassification risk.	Low

Qualitative Synthesis of Results

Elevated prehospital lactate was consistently associated with an increased likelihood of early operative or resuscitative interventions [1-4]. Lactate predicted emergency surgery, interventional radiology, and resuscitative care, including in normotensive patients [2]. Compared with traditional physiological parameters, lactate demonstrated superior or complementary predictive performance, with higher sensitivity and discriminative ability [1,2,4]. Studies defining early outcomes within six hours provided the strongest evidence for lactate’s utility in immediate operative decision-making [1,2]. Clinically relevant thresholds were reported, with levels ≥2.5 mmol/L associated with higher intervention risk, and a dose-response relationship observed across studies [1-4]. The predictive value was consistent across trauma subgroups, injury mechanisms, and transport modalities, supporting the generalizability of prehospital lactate as a triage adjunct.

Discussion

This systematic review synthesized the available evidence assessing prehospital POC lactate measurement as a predictor of early operative or emergency intervention in trauma patients. Across the included studies, elevated prehospital lactate was consistently associated with a higher likelihood of early invasive management, including emergency surgery, interventional radiology, and advanced resuscitative care. Despite heterogeneity in study design, patient populations, and outcome definitions, the direction of association was uniform, supporting lactate as a clinically relevant marker of injury severity and occult hypoperfusion in the prehospital trauma setting.

The findings suggest that prehospital lactate provides prognostic information that extends beyond traditional physiological parameters, particularly in patients who may appear hemodynamically stable on initial assessment. This characteristic is especially relevant in trauma care, where compensatory mechanisms can mask early shock and delay definitive management. Studies focusing on short outcome windows demonstrated the strongest association between lactate elevation and early intervention, underscoring the value of lactate measurement for identifying patients at risk of time-critical surgical or procedural needs rather than later clinical deterioration.

Importantly, all available evidence to date remains observational, limiting conclusions regarding the impact of lactate-guided decision-making on clinical outcomes. While elevated lactate appears to function as a reliable risk stratification tool, there is insufficient evidence to determine whether its routine use alters operative timing, resource utilization, or patient-centered outcomes. This highlights a persistent gap between diagnostic capability and actionable prehospital interventions and underscores the need for prospective studies evaluating lactate-informed triage and resuscitation strategies.

Lactate should also be interpreted with caution, as it is a nonspecific biomarker influenced by multiple physiological and pathological factors, including stress response, pain, adrenergic stimulation, fluid administration, and underlying comorbidities. Consequently, prehospital lactate is best viewed as an adjunct to, rather than a replacement for, established clinical assessment and decision-making frameworks. When integrated thoughtfully into prehospital trauma evaluation, lactate measurement has the potential to enhance early risk identification and support timely escalation of care, but further high-quality research is required to define its optimal role in clinical practice.

Limitations

This review has several notable strengths, including strict inclusion criteria focused on prehospital lactate and early operative or emergency interventions, restriction of outcomes to time-bound invasive interventions, systematic assessment of study quality using QUADAS-2, and a structured qualitative synthesis allowing meaningful integration of heterogeneous findings.

The primary limitation is the small number of eligible studies (n = 4), reflecting a broader scarcity of research in this area. All included studies were observational, introducing confounding and limiting causal inference. Considerable heterogeneity existed in lactate thresholds, sampling methods, transport modalities, and outcome definitions, precluding meta-analysis. Emergency surgery was often embedded within composite endpoints rather than analyzed separately, reducing precision. Notably, no studies evaluated lactate-guided prehospital decision-making or whether acting on lactate values improves operative timing or patient outcomes. Emerging technologies, such as continuous or subcutaneous lactate monitoring, remain untested in real-world trauma populations, highlighting the early stage of translational research in this field.

## Conclusions

Prehospital POC lactate appears to be a reliable predictor of early operative or emergency intervention in trauma patients, including those without obvious hypotension. Evidence is limited by the small number of studies, observational designs, and variability in outcome definitions. Prospective research is needed to evaluate lactate-guided prehospital strategies and their impact on surgical decision-making, resource utilization, and patient outcomes. Addressing this gap could enhance trauma triage and improve early surgical care.

## Appendices

Appendix 1

Search Strategies for Included Databases

**Pubmed**: ( ("Wounds and Injuries"[Mesh] OR trauma*[tiab] OR injur*[tiab])) AND ( ("Emergency Medical Services"[Mesh]   OR "Prehospital Care"[Mesh]  OR prehospital[tiab] OR pre-hospital[tiab] OR ambulance*[tiab] OR EMS[tiab] OR "air medical"[tiab]  OR HEMS[tiab] ) AND ( ("Lactic Acid"[Mesh] OR lactate[tiab] OR "blood lactate"[tiab] OR "serum lactate"[tiab])) AND ( (surgery[tiab]  OR operative[tiab] OR "operative intervention"[tiab] OR "emergency surgery"[tiab] OR laparotomy[tiab] OR thoracotomy[tiab] OR "interventional radiology"[tiab] OR "resuscitative care"[tiab]   OR transfusion[tiab])) Cochrane: ( ("Wounds and Injuries"[Mesh] OR trauma*[tiab] OR injur*[tiab])) AND ( ("Emergency Medical Services"[Mesh]   OR "Prehospital Care"[Mesh]  OR prehospital[tiab] OR pre-hospital[tiab] OR ambulance*[tiab] OR EMS[tiab] OR "air medical"[tiab]  OR HEMS[tiab] ) AND ( ("Lactic Acid"[Mesh] OR lactate[tiab] OR "blood lactate"[tiab] OR "serum lactate"[tiab])) AND ( (surgery[tiab]  OR operative[tiab] OR "operative intervention"[tiab] OR "emergency surgery"[tiab] OR laparotomy[tiab] OR thoracotomy[tiab] OR "interventional radiology"[tiab] OR "resuscitative care"[tiab]   OR transfusion[tiab])) **Web of Science**: The Web of Science Core Collection was searched using a structured topic-based strategy (TS), which includes titles, abstracts, and author keywords. Search terms related to trauma included “trauma” and “injury.” Prehospital care was defined using the terms “prehospital,” “pre-hospital,” “ambulance,” “emergency medical services,” “EMS,” “HEMS,” and “air medical.” Lactate-related terms included “lactate,” “lactic acid,” “blood lactate,” and “serum lactate.” Outcome-related terms comprised “surgery,” “operative,” “operative intervention,” “emergency surgery,” “laparotomy,” “thoracotomy,” “interventional radiology,” “transfusion,” and “resuscitative care.” These terms were combined using Boolean logic to identify studies examining the association between prehospital lactate measurement and early operative or emergency interventions in trauma patients. Searches were conducted from database inception to the date of the final search without restrictions on publication year or language. **Scopus**: A comprehensive literature search was conducted in Scopus using a combination of keywords related to trauma, prehospital care, lactate measurement, and emergency operative or resuscitative interventions. The search was performed in the Title, Abstract, and Keywords fields. Trauma-related terms included “trauma” and “injury”. Prehospital care was captured using the terms “prehospital,” “pre-hospital,” “ambulance,” “emergency medical services,” “EMS,” “air medical,” and “HEMS.” Lactate-related terms included “lactate,” “lactic acid,” “blood lactate,” and “serum lactate.” Outcome-related terms encompassed “surgery,” “operative,” “operative intervention,” “emergency surgery,” “laparotomy,” “thoracotomy,” “interventional radiology,” “transfusion,” and “resuscitative care.” These terms were combined using Boolean operators (AND/OR) to identify studies evaluating prehospital lactate in relation to early operative or emergency interventions in trauma patients. No date or language restrictions were applied.
